# The nationwide ‘ZNIEFF’ inventory in France: an open dataset of more than one million species data in zones of high ecological value

**DOI:** 10.3897/BDJ.10.e71222

**Published:** 2022-03-04

**Authors:** Fanny Lepareur, Mathieu Manceau, Yorick Reyjol, Julien Touroult, Solène Robert, Frédéric Vest, Arnaud Horellou, Laurent Poncet

**Affiliations:** 1 UMS Patrimoine Naturel (PatriNat) (OFB-CNRS-MNHN), Paris, France UMS Patrimoine Naturel (PatriNat) (OFB-CNRS-MNHN) Paris France

**Keywords:** key biodiversity areas, trigger species, natural heritage survey, terrestrial and marine biodiversity

## Abstract

**Background:**

In France, a ‘natural zone of ecological, faunistic or floristic value’ (Zone Naturelle d'Intérêt Écologique, Faunistique et Floristique - ZNIEFF) is a natural area, regionally known for its remarkable ecological characteristics. The ZNIEFF inventory is a naturalist and scientific survey programme launched in 1982 by the Ecology Ministry, with support from the French National Museum of Natural History (MNHN).

**New information:**

This paper describes the ZNIEFF national dataset, which comprises 1,013,725 data for various animal (38%), plant (59%) and fungal (2%) species in terrestrial and marine zones (May 2020). A total of 19,842 sites throughout continental France. as well as in the overseas Departments and territories (Guadeloupe, Martinique, Mayotte, La Réunion, French Guiana, Saint-Martin, Saint-Barthélemy and Saint-Pierre-et-Miquelon), are included in the ZNIEFF dataset (May 2020). This dataset is now available in open access.

All data were collected by skilled naturalists using professional protocols over almost 40 years. They consist mainly of observations of rare, threatened or endemic species, all validated by regional experts. Data are updated twice a year after national validation in both national (INPN-OpenObs) and global (GBIF) biodiversity web platforms. Some of the observed species, the so-called ‘trigger species’ or ‘determinant’ species, are of central interest for a site to be designated a ZNIEFF (zone of high ecological value). This concerns more than 35,000 taxa, mainly angiosperms, insects, fungi, birds and fish.

## Introduction

In France, a ‘natural zone of ecological, faunistic or floristic value’ (Zone Naturelle d'Intérêt Écologique, Faunistique et Floristique - ZNIEFF) is a natural area, regionally known for its remarkable ecological characteristics (Horellou et al. 2017). The ZNIEFF inventory is a naturalist and scientific survey programme launched in 1982 by the Ecology Ministry, with support from the French National Museum of Natural History (MNHN). The ZNIEFF programme was then integrated into French environmental law on 8 January 1993 (Art. 23, Law no. 93-24). The regulatory framework for ZNIEFFs was reinforced over time through several circulars, decrees and laws. The aim of this inventory is to use naturalist's knowledge obtained in the field to determine the location of natural sites (terrestrial and marine) with high ecological value in France and to assist in land-planning issues. This programme is conceptually similar to the Key Biodiversity Areas (KBA) initiative developed on the international level (IUCN 2016), but with a more regional and national scale of issues.

The ZNIEFF inventory is included in the National Inventory of Natural Heritage (INPN) (Poncet 2013) as part of the national information system for sharing observational data on biodiversity in France (Système d’Information de l'Inventaire du Patrimoine naturel, SINP). The ZNIEFF species dataset is, therefore, now available in open access in both national (INPN-OpenObs) and global (GBIF) biodiversity web platforms.

## General description

### Purpose

The inclusion of a site in the ZNIEFF inventory is based on the presence of a species or associations of species of high ecological value (named ‘determinant species’ in the ZNIEFF terminology). The presence of at least one determinant species makes it possible to create a ZNIEFF. The data on remarkable species and habitats in the area are collected, analysed and synthesised (Horellou et al. 2017). There are two types of ZNIEFF. Type I ZNIEFFs are ecologically-homogeneous areas, defined by the presence of species, associations of species or habitats that are rare, remarkable or characteristic of the regional natural heritage. Type II ZNIEFFs usually correspond to larger areas that integrate functional and landscaped natural zones. Type I ZNIEFFs can be included in type II ZNIEFFs (Horellou et al. 2014). If all the determinant species become extinct in an area, the site loses its official designation as a ZNIEFF.

The Regional Scientific Council for Natural Heritage (CSRPN) scientifically validates the naturalist surveys (sites and species) in each administrative region in close relationship with the regional services of the Ministry of Ecology and their scientific secretariat ‘ZNIEFF’, while the French National Museum of Natural History is in charge of the national consistency.

A ZNIEFF is not a ‘protected area’ per se, but rather a survey of naturalistic knowledge on specific sites known to shelter remarkable species and/or characterised by remarkable environmental features (e.g. a moor on serpentine). The ZNIEFF inventory is central for prioritising issues of natural heritage, defining the national biodiversity strategy and its regional sub-strategies, creating protected areas and for generating new knowledge. It has been one of the most important items when defining the French National Strategy for the Creation of Protected Areas (SCAP). In 1993, the ZNIEFF inventory facilitated the implementation of the European Habitats Directive concerning the conservation of natural and semi-natural habitats, as well as wild fauna and flora, enabling the constitution of an operational Natura 2000 network of sites. Finally, it constitutes a decision-making tool and is frequently used for environmental studies related to land planning. Generally speaking, the ZNIEFF inventory has been one of the main factors in collecting and gathering naturalist's knowledge on the national level for almost 40 years.

### Additional information

The notion of ‘determinance’ is the cornerstone of the ZNIEFF inventory and literally means ‘which determines the value and justifies the choice of the geographic area’. Each ZNIEFF must necessarily contain at least one ‘determinant’ species (also called ‘trigger’ species in scientific literature) to be considered as such. The determining characteristic is the intrinsic value of the species (e.g. localised, threatened on the regional, national or international level, endemic, at the limit of the range etc.), combined with the particular conditions of the site (notably, the importance of the species population in the region with respect to its presence in other regions and its global geographic distribution). In addition to these determinant species, the ZNIEFF inventory also takes into account determinant habitats, which contribute to the selection of the area on their own value or that of the species they shelter. Data related to other (non-determinant) species are also collected during field surveys and generally included in the dataset, given that it is useful for understanding the global ecological functioning of the ZNIEFF or to anticipate global anthropogenic threats or more local impacts (e.g. extension of the range of species due to climate change or urbanisation). Only data concerning species (not habitats) will be presented in this datapaper, as habitat data do not yet benefit from viewing and downloading tools at the national level.

There is a particular case of species with ‘confidential distribution’. This concerns a limited number of species in a given region that are particularly ‘sensitive’, i.e. subject to harmful human activity and for which the availability of data is likely to increase the likelihood of the harmful activity occurring (Chapman and Grafton 2008). This may concern species which are threatened, rare and/or of high heritage interest and for which the dissemination of information represents (in the regional context) a risk of targeted destruction. This dissemination of information could also seriously affect the conservation status of the population of a species or the habitat that shelters it. The confidentiality of a species must, however, remain exceptional and is assessed on a case-by-case basis on the regional level by the scientific council. The corresponding data are excluded from the dataset on the INPN website and the GBIF portal.

In addition to this notion of confidential data specific to the ZNIEFF programme, there is also a national programme on 'sensitive data'. This programme is recent and aims to harmonise and implement blurring of what is called sensitive/confidential/restricted data in some French datasets, including the ZNIEFF inventory. Data considered sensitive can be blurred more widely than a zone, i.e. to the Municipality, the Department, a grid cell etc. This is mentioned in the 'informationWithheld' and 'dataGeneralizations' fields of the ZNIEFF dataset.

## Project description

### Personnel

In order to guarantee the consistency of the information, data are collected using a common framework (Horellou et al. 2017) defined jointly by a scientific and technical coordination team, based in the French National Museum of Natural History (UMS PatriNat) and the Ecology Ministry. The observations are transmitted by the wide-ranging naturalist network in France, including independent professional and amateur naturalists, associations for nature studies and protection, public institutions, contractors (private sector), learned societies etc. The Regional Scientific Councils for Natural Heritage (CSRPN) validate the local inventories in close conjunction with the regional services of the Ecology Ministry and their scientific secretariat 'ZNIEFF' before transmission to the MNHN for validation to ensure its technical and scientific consistency. The data are then sent to the ZNIEFF database via a web application specifically designed for this purpose.

### Funding

The Ecology Ministry coordinates the regional services and provides financial support for the annual operation of the ZNIEFF programme.

## Sampling methods

### Sampling description

Sampling is focused on areas of high biodiversity identified at the regional scale. These zones (19,842 registered as ZNIEFFs on May 2020; Table 1) are pre-identified, based on the knowledge of field naturalists, often through the primary study of vegetation, but occasionally also on abiotic characteristics (e.g. a moor on a serpentine substrate). The subsequent inventory of specific taxa of interest on these sites helps to confirm, or not, their value for conservation. ZNIEFF data come mainly from direct field observations, but can also rely on literature sources, collections and other databases of direct observations. A great variety of sampling methods and protocols are used to collect data, depending on the environment and the taxon concerned.

All figures given in this publication, including illustrations, were calculated in May 2020.

### Quality control

The dataset presented in this publication is managed by UMS PatriNat (OFB/CNRS/MNHN), the unit also responsible for the National Inventory of Natural Heritage (INPN). The INPN is part of the SINP, i.e. the French national information system for sharing observational data on biodiversity. This information system guarantees the traceability of data, authorship and normalised standards of data and metadata.

Before integration and dissemination on the INPN website, a series of validation checks are systematically performed (Jomier et al. 2019). The first category of checks consists of validating compliance with standard data and metadata formats, i.e. all mandatory fields completed, use of required formats, repositories (including the geographic and taxonomic repositories), classifications and lists of values. The second category of checks consists of validating the consistency (i.e. the absence of logical incompatibility) within the data, within the metadata and between the data and the metadata. As an example, the observation start date must be less than or equal to the observation end date. ZNIEFF data are updated two times a year.

The INPN provides the data directly to the GBIF portal in order to make the data available at the international level.

All taxa are identified by experienced naturalists prior to the validation by the regional scientific councils (CSRPN). The dataset producers are responsible for the reliability of the identification.

## Geographic coverage

### Description

**Figure 1.**
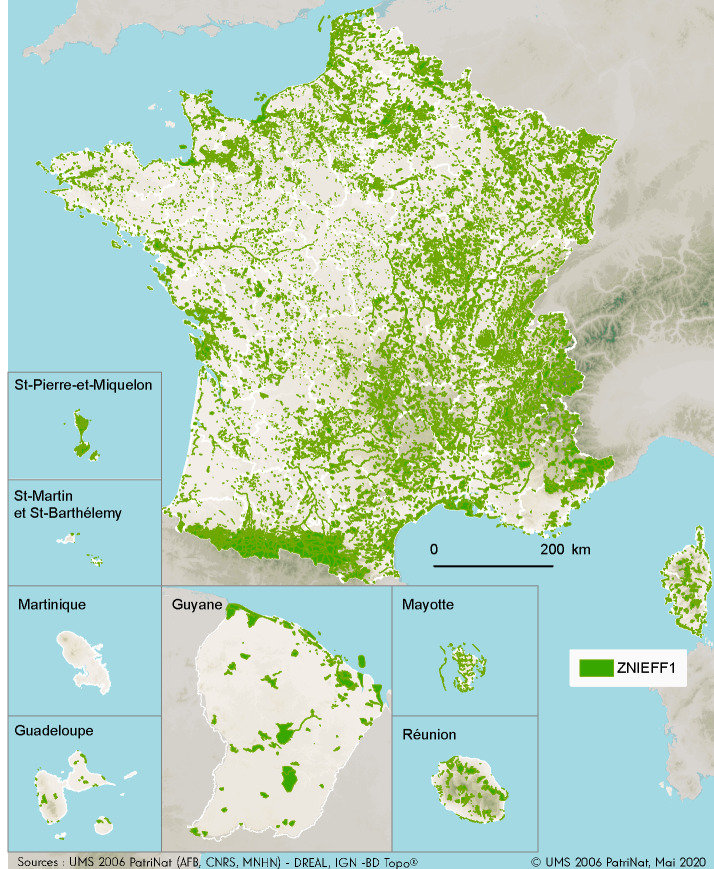
Map of the Type I ZNIEFFs (continental and marine).

The inventory covers the marine, terrestrial and freshwater environments of all administrative regions of continental France and its overseas territories (five overseas Departments: Guadeloupe, Martinique, Mayotte, La Réunion, French Guiana and three overseas territories: Saint-Martin, Saint-Barthélemy and Saint-Pierre-et-Miquelon) (Fig. 1).

The description and mapping of the area are specified by the ZNIEFF method and the geographic boundaries of each ZNIEFF must have a scientific basis (ecological, consistent with the heritage and functional value of the area, with particular reference to criteria for the distribution of habitats, vegetation, geomorphology, land use etc.).

Maps can be visualised on the INPN website (https://inpn.mnhn.fr/viewer-carto/espaces). The map of each ZNIEFF can be accessed independently (e.g. https://inpn.mnhn.fr/zone/znieff/930012348).

Geographic data can be downloaded in the form of GIS layers (three available formats) and web services (WMS and WFS):

Type I – continental ZNIEFF: https://inpn.mnhn.fr/telechargement/cartes-et-information-geographique/inv/znieff1

Type II – continental ZNIEFF: https://inpn.mnhn.fr/telechargement/cartes-et-information-geographique/inv/znieff2

Type I – marine ZNIEFF: https://inpn.mnhn.fr/telechargement/cartes-et-information-geographique/inv/znieff1_mer

Type II – marine ZNIEFF: https://inpn.mnhn.fr/telechargement/cartes-et-information-geographique/inv/znieff2_mer

## Taxonomic coverage

### Description

The ZNIEFF inventory focuses on all fauna, flora and fungal taxa, with a special emphasis on rare, threatened or endemic taxa of the taxonomic groups which are best studied by naturalists. The considered taxa are those considered indigenous to French territories and they are examined on the specific and infra-specific levels (Horellou et al. 2014).

The taxonomy complies with the standards of the national repository TAXREF for the fauna, flora and fungi of continental France and the overseas Departments and territories (Gargominy et al. 2019). TAXREF assigns a unique, unambiguous and (wherever possible) consensual scientific name to all species occurring in these territories. A new version of TAXREF is published yearly on the INPN website (link above) and the GBIF portal (https://www.gbif.org/dataset/0e61f8fe-7d25-4f81-ada7-d970bbb2c6d6).

**Figure 2.**
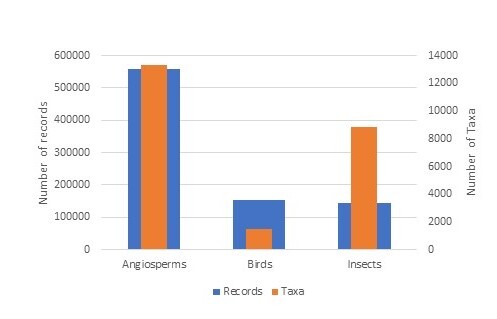
Taxonomic coverage of the ZNIEFF inventory (number of taxa and data records per group) for 'taxonomic' groups that have more than 100,000 data records.

**Figure 3.**
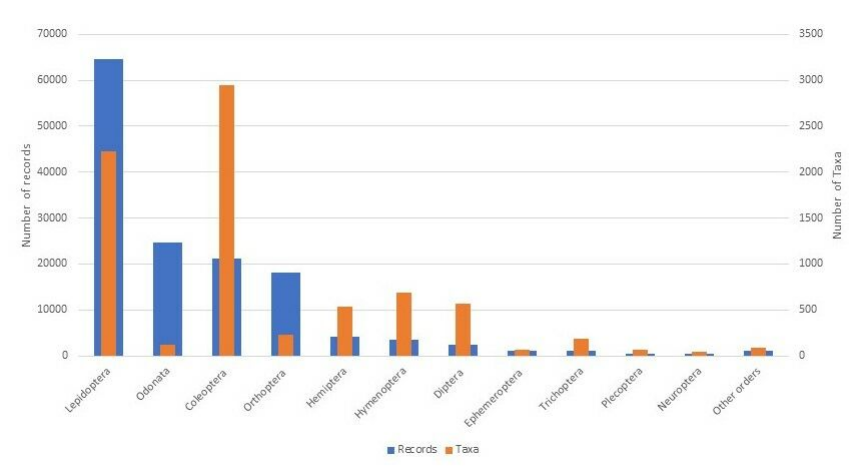
Taxonomic coverage of the ZNIEFF inventory (number of taxa and data records per order) for insects.

**Figure 4.**
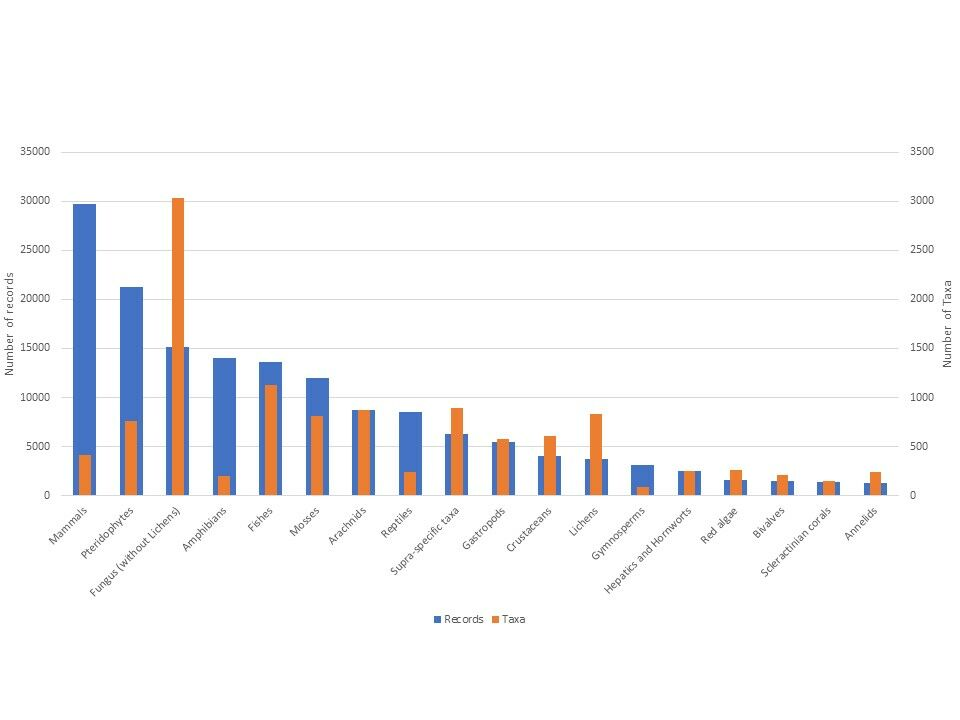
Taxonomic coverage of the ZNIEFF inventory (number of taxa and data records per group) for 'taxonomic' groups that have less than 100,000 and more than 1,000 data records.

More than 35,000 taxa (species and subspecies) have been inventoried in ZNIEFFs to date, which represents approximately 19% of the number of species currently listed in France (INPN 2019). The taxa targeted by the ZNIEFF inventory usually have high conservation value at the national and supra-national levels (taxa which are geographically localised, endemic, at the limit of their range, threatened on a regional, national or international level etc.). The taxonomic groups with the highest number of taxa inventoried are, in decreasing order, angiosperms (13,312), insects (8,823), fungi (3,033), birds (1,503) and fish (1,128) (Table 2, Figs 2, 3, 4). These groups have a significant percentage of endemic or subendemic taxa and species assessed as threatened according to the French Red List (Table 2).

**Figure 5.**
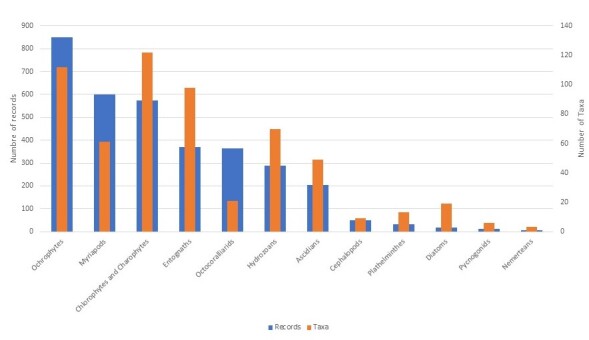
Taxonomic coverage of the ZNIEFF inventory (number of taxa and data per group) for 'taxonomic' groups that have less than 1,000 data records.

Three taxonomic groups have more than 100,000 records, namely angiosperms (559,081), birds (153,047) and insects (144,237) (Fig. 2). Amongst the group of insects, the orders Lepidoptera, Odonata and Coleoptera are the most represented in the dataset (Fig. 3). Five groups have more than 10,000 data records (mammals, pteridophytes, fungi, amphibians, fish and mosses) (Fig. 4). The remaining groups have less than 1,000 records (Fig. 5).

## Temporal coverage

### Notes

The data span the years 1757 to 2019.

The ZNIEFF inventory was officially launched in 1982, but data prior to this date have been taken into account to justify the ecological value of some areas. There were two inventory phases (i.e. ‘generations’ according to the ZNIEFF terminology). The first generation took place between 1982 and 1995, while the second generation lasted from 1995 to 2014 (Horellou et al. 2017). These phases corresponded to important methodological changes. Since 2017, the ZNIEFF inventory is continuously updated, that is to say, there are now no more ‘generations’.

**Figure 6.**
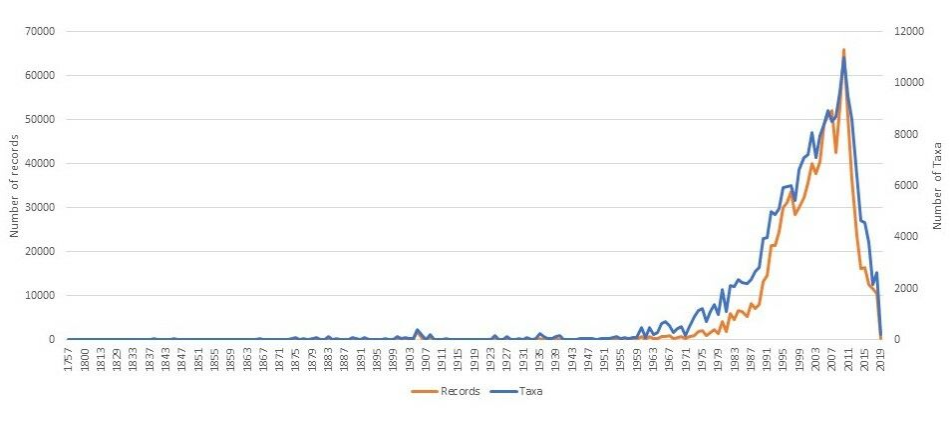
Temporal distribution of the ZNIEFF dataset for species from 1757 to 2019.

The effort put into field surveys and data collection in regional information systems was very important until 2010 (Fig. 6). Following this tremendous collection effort, the subsequent sampling effort on the regional level became more qualitative, mainly dedicated to determinant species and taking into account the territorial specificities in terms of natural habitats and naturalistic networks. Despite the drop in annual record count observed in Fig. 6, new data are incorporated into the dataset every year, with national validation taking place twice a year.

## Usage licence

### Usage licence

Other

### IP rights notes

This project is licensed under a Creative Commons Attribution 4.0 International Licence (CC BY 4.0).

## Data resources

### Data package title

Données d'occurrences Espèces issues de l'inventaire des ZNIEFF

### Resource link

https://doi.org/10.15468/ikshke

### Alternative identifiers

http://ipt.gbif.fr/resource?r=inpn_inventaire_znieff

### Number of data sets

1

### Data set 1.

#### Data set name

Données d'occurrences Espèces issues de l'inventaire des ZNIEFF

#### Data format

Darwin Core Archive

#### Number of columns

32

#### Character set

Unicode (UTF-8)

#### Download URL

http://ipt.gbif.fr/resource?r=inpn_inventaire_znieff

#### Description

The description of the ZNIEFF dataset for species is also available on the INPN website (https://inpn.mnhn.fr/espece/jeudonnees/7907). The ZNIEFF dataset can be downloaded from the INPN-OpenObs portal and downloaded from the GBIF portal (data as csv files or a Darwin Core Archive). It is important to mention that the X, Y coordinates correspond with the centroid of the zones.

**Data set 1.** 

Column label	Column description
id	The unique identifier of the Occurrence.
modified	The most recent date on which the resource was changed (YYYY).
language	In English and French (en | fr).
datasetID	The identifier for the dataset.
institutionCode	The name in use by the institution having custody of the information referred to in the record.
basisOfRecord	The specific nature of the data record.
informationWithheld	The field indicates whether the taxon is regionally sensitive, i.e. the data should not be disseminated specifically to avoid harm to the taxon. This is a different level of sensitivity from that explained in the General description of the datapaper. This regional sensitivity implies blurred data.
dataGeneralizations	When the data is considered sensitive (cf. informationWithheld), this field indicates the blurring applied to the data.
occurrenceID	The unique identifier of the Occurrence (=id).
occurrenceRemarks	Indication if the species is determinant for this ZNIEFF. The data field is empty otherwise.
recordedBy	A list of names of people, groups, bibliographic reference or organisations responsible for recording the original Occurrence. This list is not standardised due to the old existence of this programme.
occurrenceStatus	A statement about the presence of a taxon in a ZNIEFF.
associatedReferences	The identifier and the full name of the ZNIEFF in which the taxon occurs.
eventID	The identifier of the ZNIEFF in which the taxon occurs.
eventDate	The year or the interval when the observation of the taxon was recorded (YYYY or YYYY/YYYY).
country	The name of the country in which the Location occurs.
countryCode	The standard code for the field stateProvince in which the Location occurs.
stateProvince	The name of the French territories (Metropolitan France and each overseas territories) in which the Location occurs.
county	The standard code of French Department in which the ZNIEFF occurs.
municipality	The full name and the postal code of the Municipality in which the ZNIEFF occurs (Postal code|Name).
locationRemarks	When the data is considered sensitive (cf. informationWithheld), this field indicates that the location has been blurred. The decimalLatitude and decimalLongitude fields are then the centroid of the nearest grid cell.
decimalLatitude	The geographic latitude of the centroid of the ZNIEFF (in decimal degrees, using the spatial reference system WGS84).
decimalLongitude	The geographic longitude of the centroid of the ZNIEFF (in decimal degrees, using the spatial reference system WGS84).
coordinateUncertaintyInMetres	The horizontal distance (in metres) from the given decimalLatitude and decimalLongitude describing the smallest circle containing the whole of the Location.
identificationVerificationStatus	Indicator of the extent to which the taxonomic identification has been verified to be correct. From a scientific point of view, the taxonomic identification has been verified, but it still lacks technical controls.
taxonID	The unique identifier for the taxon (scientificName) according to TAXREF (=CD_NOM in TAXREF).
scientificName	The full scientific name, with authorship and date information.
nameAccordingTo	According to the national repository TAXREF for the fauna, flora and fungi of continental France and the overseas Departments and territories (with the last version used).
kingdom	The full scientific name of the kingdom in which the taxon is classified.
class	The full scientific name of the class in which the taxon is classified.
order	The full scientific name of the order in which the taxon is classified.
family	The full scientific name of the family in which the taxon is classified.

## Additional information

When using the data from the ZNIEFF inventory, synthesised species data (see Data Resources) should be associated with the perimeters of the zones (see the Geographic coverage section above). The X, Y coordinates in the dataset, downloadable from the GBIF portal, correspond with the centroids of the zones. It is, therefore, necessary to link the synthesised data and the perimeters with the common field, which is the identifier of the ZNIEFF ('Event ID' in the dataset and 'NM_SFFZN' in GIS files).

These data can be used for all types of analyses and studies on key biodiversity areas, protected areas and ecological networks. They can also be used for maps of rare species, distribution atlases etc. These data alone are not suitable for precise monitoring of populations nor for establishing temporal trends of distribution.

Caution is advised in that there may be differences in inventory intensity and species groups studied depending on the region. Comparison of species occurrence (and even more so of richness) amongst sites is more relevant within a given region and should be considered with caution at a national level.

## Figures and Tables

**Table 1.** Number of sites registered in the ZNIEFF inventory according to the different categories.

	Type I	Type II	Total
Continental ZNIEFFs	17,368	2,248	19,616
Marine ZNIEFFs	132	94	226
Total	17,500	2,342	19,842

**Table 2.** Taxonomic coverage of the ZNIEFF inventory (species and subspecies) per ‘taxonomic’ groups arranged in alphabetical order. The Table presents the number of records, the total number taxa, the number of endemic and sub-endemic taxa and the number of taxa assessed as threatened on the national level according to the French IUCN Red List (threatened categories for IUCN: VU, EN and CR).

‘Taxonomic’ groups	Number of records	Total number of taxa	Number of endemic and sub-endemic taxa (% of the total number of taxa is given in parentheses)	Number of threatened taxa (% of the total number of taxa is given in parentheses)
**Animalia**:	**387,441**	**15,286**		
Amphibians	14,021	195	31 (15.9%)	13 (6.7%)
Annelids	1,276	242		
Arachnids	8,766	875	24 (2.7%)	
Ascidians	205	49		
Birds	153,047	1,503	44 (2.9%)	261 (17.4%)
Bivalves	1,444	208	1 (0.5%)	
Cephalopods	49	9		
Crustaceans	3,990	607	99 (16.3%)	45 (7.4%)
Entognaths	369	98	73 (74.5%)	
Fish	13,567	1,128	41 (3.6%)	28 (2.5%)
Gastropods	5,489	575	85 (14.8%)	
Hydrozoans	289	70		
Insects	144,237	8,823	412 (4.7%)	52 (0.6%)
Mammals	29,700	409	31 (7.6%)	24 (5.9%)
Myriapods	601	61	5 (8.2%)	
Nemerteans	7	3		
Octocoralliarids	363	21		
Plathelminthes	33	13	3 (23.1%)	
Pycnogonids	13	6		
Reptiles	8,564	238	24 (10.1%)	28 (11.8%)
Scleractinian corals	1,411	153		14 (9.2%)
**Plantae**:	**600,220**	**15,616**		
Angiosperms	559,081	13,312	1,058 (7.9%)	770 (5.8%)
Chlorophytes and Charophytes	574	122		
Gymnosperms	3,071	89		2 (2.2%)
Hepatics and Hornworts	2,546	254		
Mosses	12,017	809	1 (0.1%)	
Pteridophytes	21,291	765	64 (8.4%)	120 (15.7%)
Red algae	1,640	265		
**Fungi**:	**18,877**	**3,862**		
Fungus (without Lichens)	15,149	3,033		
Lichens	3,728	829		
**Chromista**:	**870**	**131**		
Diatoms	19	19		
Ochrophytes	851	112		
**Supra-specific taxa**	**6,317**	**889**		
**TOTAL**	**1,013,725**	**35,784**		
